# Effects of fermented feed on growth performance, serum biochemical indexes, antioxidant capacity, and intestinal health of lion-head goslings

**DOI:** 10.3389/fvets.2023.1284523

**Published:** 2023-11-02

**Authors:** Zhiqi Fu, Na Ao, Xiaoen Liang, Jinhuang Chen, Yuchuan Wang, Qing Wang, Jing Fu, Chunpeng Liu, Lizhi Lu

**Affiliations:** ^1^College of Animal Science and Technology, Zhongkai University of Agriculture and Engineering, Guangzhou, Guangdong, China; ^2^Innovative Institute of Animal Healthy Breeding, Zhongkai University of Agriculture and Engineering, Guangzhou, Guangdong, China; ^3^College of Life Sciences, Jiaying University, Meizhou, China; ^4^State Key Laboratory for Managing Biotic and Chemical Threats to the Quality and Safety of Agro-Products, Institute of Animal Husbandry and Veterinary Science, Zhejiang Academy of Agricultural Sciences, Hangzhou, China

**Keywords:** fermented feed, lion-head goslings, growth performance, antioxidant capacity, intestinal health

## Abstract

**Introduction:**

The aim of this study was to evaluate the effects of fermented feed on growth performance, antioxidant indexes and intestinal health in lion-head goslings.

**Methods:**

288 male lion-head goslings (one-day-old) were randomly divided into four groups (6 replicates per group, 12 samples per replicate): control group (basal diet) and fermented feed (FF) groups (basal diet supplemented with 2.5, 5.0 and 7.5% FF, respectively). The experimental period lasted 28 days.

**Results:**

The results showed that 5.0 and 7.5% FF groups decreased feed conversion rate (FCR) when compared with the control group (*p* < 0.05). The 5.0% FF group reduced the activity of alkaline phosphatase (ALP) and lactate dehydrogenase (LDH) in serum; while the 7.5% FF group decreased the concentration of total cholesterol (TC), ALP and LDH activity (*p* < 0.05). Furthermore, the 7.5% FF group significantly increased total antioxidant capacity (T-AOC) in serum (*p* < 0.05); 2.5% and 5.0% FF groups significantly increased glutathione peroxidase (GSH-Px) in serum (*p* < 0.05); all FF groups increased the activity of superoxide dismutase (T-SOD) in serum (*p* < 0.05). For intestinal health, the villous height and villi/crypt ratio in jejunum were increased in all FF groups, but crypt depth was decreased (*p* < 0.05); The 5.0% FF groups enhanced T-AOC activity in jejunum (*p* < 0.05); The 2.5% and 5.0% FF groups enhanced GSH-Px activity (*p* < 0.05) in jejunum; All FF groups reduced malondialdehyde (MDA) level in jejunum (*p* < 0.05). LEfSe analysis showed that the cecum microbiota was significantly dominant in the 2.5% FF group compared to the control group including *Firmicutes*, *Lactobacillales*, *Lactobacillus*, and *Prevotella*; the flora that were significantly dominant in the 5.0% FF group compared to the control group included *Bacteroidaceae*, *Bacteroides*, *Megamonas*, and *Prevotella*; and the groups that were significantly dominant in the 7.5% FF group compared to the control group included *Bacteroidota*, *Bacteroides*, *Bacteroidaceae*, and *Ruminococcaceae*.

**Discussion:**

In summary, dietary FF supplementation improved growth performance, serum biochemical parameters and antioxidant capacity of lion-head goslings, as well as improved jejunal tissue morphology and optimized intestinal flora structure. In particular, the FF addition at a dose of 7.5% was relatively more effective for lion- head goslings.

## 1. Introduction

With the rapid development of large-scale and intensive livestock farming, livestock diseases have become increasingly complex and diverse and livestock intestinal health problems have become increasingly prominent. The healthy development of animal husbandry has thus necessitated a search for alternative safe, efficient, and reasonable “green feed.” Corn and soybean meal (SBM) are widely used in animal feeds due to their high nutritional value and digestibility of organic matter. However, it has been found that corn and soybean meal contain various antinutritional factors including phytic acid, trypsin inhibitors, and soybean antigenic proteins. These substances have been shown to reduce animal growth performance and health by reducing the efficiency of diets, decreasing the digestibility and absorption rate of the feed, and by disrupting the gut microbiota of animals (1, 2). To this end, fermented feed (FF), an important component of the feed industry, has emerged as an effective strategy to reduce or replace the use of antibiotics in animal feed (3). Indeed, beneficial microorganisms in FF break down or convert macromolecules, antinutritional factors (ANFs), and toxins in the feed into substances that can be easily digested and absorbed by animals (1, 4); thus increasing the nutritional value of the feed and improving its palatability. Gut health is critical to the growth performance and health of poultry. The gut microbiota is the community of microorganisms that colonize the gut of the host organ. Microorganisms colonize the gut through a very complex process of interaction between the microorganisms and the host, which provides a site for microbial survival and evolution (5). Gut microbes are an integral part of the gastrointestinal tract and play important roles in nutrition, physiology and gut morphology. Moreover, the gut microbiota is involved in the host’s immune defence mechanisms against pathogens (6). Beneficial microorganisms in fermented feeds, such as lactic acid bacteria, reduce gut pH by producing organic acids and inhibit colonization of gut pathogens through antagonistic activity, competitive exclusion and bacteriocin production (7). Numerous Studies have shown that the use of FF in poultry production can improve growth performance, antioxidant function, regulate serum biochemical indexes, improve immunity, and maintain intestinal microecological balance (8–10). The use of FF can also reduce the cost of feeding and improve overall economic efficiency. Additionally, due to the wide range of raw materials contained in FF its use has the potential to reduce the waste of resources and environmental pollution associated with traditional feeds (11). Therefore, FF has broad application prospects in livestock and poultry production.

As herbivorous waterfowl, geese have a greater advantage than other poultry species in digesting and utilizing dietary fiber, tolerating roughage and utilizing straw and silage to partially replace concentrates in the goose diet, thus reducing costs in production. The microflora in the gut of the lion- head goose breaks down cellulose, with the final reaction producing volatile fatty acids, ammonia and carbon dioxide (12–14). Furthermore, the lion-head goose, the only large goose species in China, native to Rao Ping County in Chaozhou City, Guangdong Province, is a protected agricultural resource. These geese are characterized by their large size and fast growth rate (15), Lion-head goose meat is of excellent quality, it is rich in essential amino acids needed for human growth and development, and rich in monounsaturated fatty acids, linolenic acid and arachidonic acid, and contains more linolenic and linoleic acid than chicken, Popular with consumers (16). Popular with consumers. However, at present, the entire lion head goose breeding is still in the initial stage, especially in the lion-head goose brood stage, there are many problems, due to the incomplete development of the intestinal tract of the goslings, poor environmental factors and inappropriate feeding and management conditions, such as the temperature, humidity and other stresses, so that the lion-head goslings intestinal health and other problems are becoming increasingly prominent. Serious constraints on the feeding management of lionhead geese in the brood stage, affect the economic benefits of lionhead goose breeding. At present, there have been many studies on the application of fermented feed to chickens, ducks and other poultry. To date, the application of FF in the breeding of lion-head geese has rarely been reported. Therefore, in this study, 1 to 28-day-old lion-head goslings were fed different doses of FF to assess changes to growth performance, serum biochemical parameters, antioxidant capacity and intestinal health compared to a basic diet. The results of this study may provide a theoretical basis for the application of FF in lion-head goose breeding.

## 2. Materials and methods

### 2.1. Fermented feed preparation

The FF was developed in-house. The fermentation process was as follows: fermentation substrate (50% bran, 40% ultrapure water, 5% corn flour, 3% molasses) was mixed with liquid fermented *Lactobacillus plantarum* (1 × 10^9^ cfu/mL, 2%), placed in fermentation bags, and fermented at 37°C for 7 days. The final product has an even yellow-brown color, is weakly acidic (pH = 4.1), and has a soft texture. The nutritional composition of the FF was determined as follows: 45.05% moisture, 7.40% crude protein, 2.07% crude fat, 3.52% crude fiber, 2.48% crude ash, 0.05% calcium, and 0.48% total phosphorus.

### 2.2. Experimental design and diet

The goslings were provided by “Rao Ping Lion-headed Goose Science and Technology Institute, Chaozhou City, Guangdong Province”. In the experiment, 288 1-day-old healthy male lion-head goslings with similar body weights were selected and randomly divided, under the experimental design (one-way ANOVA), into 4 groups with 6 replicates per group and 12 animals per replicate. FF was added at a concentration of 0 (the control group), 2.5, 5.0, and 7.5% into the basic diet of the 4 groups respectively; the experimental period was set to 28 d. The basic diet used in the experiment was formulated in accordance with the Nutrient Requirements of Poultry (NRC) (1994) and that of the lion-head goose production industry in Guangdong Province. The composition and nutritional levels of the feed are listed in Table 1.

**Table 1:** Composition of basal diets.

Items	Content/%
Ingredient	
Corn	58.00
Soybean meal	24.00
Bran	6.50
Rice bran	8.20
CaHPO4	1.20
Limestone	1.10
Premix^a^	1.00
Total	100.00
Nutrient levels^b^	
Metabolizable energy (MJ/kg)	11.50
Crude protein	17.46
Ether extract	4.07
Calcium	0.85
Phosphorus	0.83
Lysine	0.89

a The premix provided the following per kg of diets: 5000 IU, VB_1_ 5.0 mg, VB_2_ 8 mg, VB_6_ 5.0 mg, VB_12_ 12 μg, VD_3_ 800 IU, VE 50 IU, VK_3_ 2.5 mg, folic acid 0.5 mg, nicotinamide 40 mg, biotin 0.3 mg, pantothenic acid 25 mg, choline 1,500 mg, Fe (as ferrous sulfate) 85.2 mg, Cu (as copper sulfate)10 mg, Zn (as zinc sulfate) 50 mg, I(as potassium iodide) 0.3 mg, Se (as sodium selenite) 0.25 mg.

b Metabolizable energy was a calculated value, while the others were measured values.

### 2.3. Feeding procedure

The brood was reared in two phases consisting of: indoors in a net bed on days 1–14, and outdoors on the ground on days 15–28. All goslings were given food and water *ad libitum* throughout the experiment. During the 28 days, immunization, sanitation, and disinfection procedures were carried out according to actual farm production standards. Welfare monitoring was performed daily to ensure the health status of the geese was maintained.

### 2.4. Sample collection and index determination

#### 2.4.1. Growth performance

Body weights (BW) of goslings were determined at the beginning and end of the trial. Feed intake was measured for each replicate, and average daily gain (ADG), average daily feed intake (ADFI), and feed conversion rate (FCR) were calculated for each replicate group at the end of the experiment.

#### 2.4.2. Serum biochemical indexes and antioxidant capacity

At the end of 28 days, all goslings were fasted for 12 h. Two goslings of similar body weight were randomly selected from each replicate for blood collection. 5 mL of blood was collected from the sub wing vein and placed at an angle until the serum was precipitated; subsequently, the blood was centrifuged at 3000 r/min for 15 min to separate the serum which was stored at −20°C for the determination of serum biochemical indexes and antioxidant indexes.

The kits were purchased from Maccura Biotechnology Co., Ltd. Total protein (TP) was determined by the bis-urea method (17), albumin (ALB) was determined by the bromocresol green method (18), triglyceride (TG) was determined by the GPO-PAP method (19), total cholesterol (TC) was determined by the CHOD-PAP method (19), alanine aminotransferase was measured using (ALT) by the alanine substrate method (20), and aspartate aminotransferase (AST) by the aspartate substrate method (20), alkaline phosphatase (ALP) was measured by NPP substrate-AMP buffer method (21), blood glucose (GLU) was measured by glucokinase method (22) and lactate dehydrogenase (LDH) enzyme was measured by lactate substrate method (23).

The kits were purchased from Nanjing Jiancheng Institute of Bioengineering. Serum total antioxidant capacity (T-AOC) was determined by ABTS rapid method, serum total superoxide dismutase (T-SOD) was determined by hydroxytoluidine method, serum glutathione peroxidase (GSH-PX) activity was determined by colourimetric method, and serum malondialdehyde (MDA) content was determined by TBA method (24, 25).

#### 2.4.3. Measurement and observation of intestinal morphological indicators

After the goslings were euthanized, a 2 cm section of the mid jejunum was taken; the contents of the intestine were then rinsed with pre-chilled phosphate buffer solution (PBS) and the samples were quickly immersed in 10% formalin and fixed. The effects of FF levels on the morphological and structural development of jejunal villi was assessed using paraffin sections, with photographs taken under by light microscopy. NDP.view2 image viewing software (Hamamatsu Photonics K.K., Japan) was used to measure villous height and crypt depth in the jejunum and to calculate the villi/crypt ratio.

#### 2.4.4. Intestinal antioxidant capacity

After the goslings were euthanized, jejunum tissues were taken and stored at −80°C. The kits were purchased from Nanjing Jiancheng Institute of Bioengineering. The total antioxidant capacity (T-AOC) of jejunum was determined by ABTS rapid method, the total superoxide dismutase (T-SOD) of jejunum was determined by hydroxytoluidine method, the activity of jejunum glutathione peroxidase (GSH-PX) was determined by colourimetric assay, and the content of jejunum malondialdehyde (MDA) was determined by TBA method (24, 25).

#### 2.4.5. Gut microbiota amplicon sequencing analysis

Post-mortem cecum segment contents were collected under sterile conditions, transferred into sterile tubes and snap frozen in liquid nitrogen prior to storage at −80°C. All samples were sent to Harbin Botai Biotechnology Co., Ltd. For DNA extraction and the quality of DNA samples was checked using a UV spectrophotometer. The target fragments of 16S rRNA V3 ~ V4 were amplified by PCR with primers 338F (5′-barcode+ACTCCTACGGGAGGCAGCA-3′) and 806R (5’-GGACTACHVGGGTWTCTAAT-3′). PCR amplification was performed using the NEB Q5 DNA high-fidelity polymerase reaction system. Libraries were built using an Illumina TruSeq Nano DNA LT Library Prep Kit, and 2 × 250 bp double-end sequencing was performed on an Illumina NovaSeq machine using NovaSeq 6,000 SP Reagent Kit (500 cycles) for qualified libraries.

#### 2.4.6. Bioinformatics analysis and data processing

The valid data were clustered into operational taxonomic units (OTUs) at a 97% similarity level using uclust (v1.2.22q) method to obtain the total number of OTUs and out relative abundance. Alpha diversity analysis (Chao1 index, observed species index, Shannon index, and Simpson index) were conducted using QIIME (v1.9.1) and the community composition of each sample was calculated according to the taxonomic level of kingdom, phylum, order, family, genus and species. Principal component analysis (PCA) was performed using R (v3.2.3) to determine the structural similarity of colonies between samples. Linear discriminant analysis effect size (LEfSe) method was used to detect taxonomic units with rich differences between groups (LDA score > 3.0).

### 2.5. Statistical analysis

One-Way ANOVA was employed for data analysis using SPSS 22.0. All groups were compared with each other using Tukey’s multiple comparison test. Significant differences were determined where *p* < 0.05 was considered significant and results were expressed as the mean and SEM.

## 3. Results

### 3.1. Effects of fermented feed on growth performance of lion-head goslings

As shown in Table 2, the FCR of 5.0 and 7.5% FF groups decreased significantly compared with the control group (*p* < 0.05), while final weight, ADG and ADFI of the FF groups did not change significantly compared with the control group (*p* > 0.05).

**Table 2:** Effect of fermented feed on growth performance of lion head goose goslings.

Items	Dietary supplemental FF level				SEM	*p* value
	0.0% FF	2.5% FF	5.0% FF	7.5% FF		
Initial BW, g	124.1	129.16	127.32	124.20	1.31	0.471
Final BW, g	1312.37	1316.32	1312.97	1441.88	23.11	0.117
ADG, g	42.44	42.40	42.34	47.06	0.82	0.100
ADFI, g	84.85	80.65	77.53	83.95	1.42	0.255
FCR	2.10^a^	1.88^ab^	1.77^b^	1.75^b^	0.48	0.023

^a,b^ Values with superscripts of different letters in the same row were significantly different (*p* < 0.05), whereas values with the same or no superscripts showed no differences (*p* ≥ 0.05).

### 3.2. Effects of fermented feed on serum biochemical indexes of lion-head goslings

According to Table 3, compared with the control group, 5.0 and 7.5% FF groups significantly decreased the activity of ALP (*p* < 0.05) in serum; the 7.5% FF group reduced the concentration of TC (*p* < 0.05) Moreover, 5.0 and 7.5% FF groups decreased LDH activity (*p* < 0.05). However, FF treatment had no significant on the levels of TP, ALB, GLO, TG, ALT, AST and GLU in serum (*p* > 0.05).

**Table 3:** Effect of fermented feeds on serum biochemical indexes of lion head goose goslings.

Items	Fermented feed supplemental level				SEM	*p* value
	0.0% FF	2.5% FF	5.0% FF	7.5% FF		
TP/(g/L)	48.22	51.10	48.96	51.48	1.11	0.703
ALB/(g/L)	9.66	9.98	9.94	10.32	0.19	0.716
GLO/(g/L)	38.24	40.4	39.94	40.26	0.95	0.864
TG/(mmol/L)	1.29	1.18	1.19	0.89	0.06	0.054
TC(mmol/L)	7.90^a^	7.60^ab^	6.69^ab^	6.43^b^	0.20	0.015
ALT/(u/L)	25.20	21.00	20.60	19.6	0.81	0.058
AST/(u/L)	42.20	41.60	41.20	42.60	1.38	0.988
ALP/(u/L)	1252.80^a^	1024.60^ab^	840.60^b^	998.40^b^	41.62	0.001
GLU/(mmol/L)	6.70	7.52	6.83	7.25	0.28	0.748
LDH/(u/L)	1479.60^a^	1208.60^ab^	1129.60^b^	950.60^b^	58.17	0.003

^a,b^ Values with superscripts of different letters in the same row were significantly different (*p* < 0.05), whereas values with the same or no superscripts showed no differences (*p* ≥ 0.05).

### 3.3. Effects of fermented feed on serum antioxidant function of lion-head goslings

**Figure 1:**
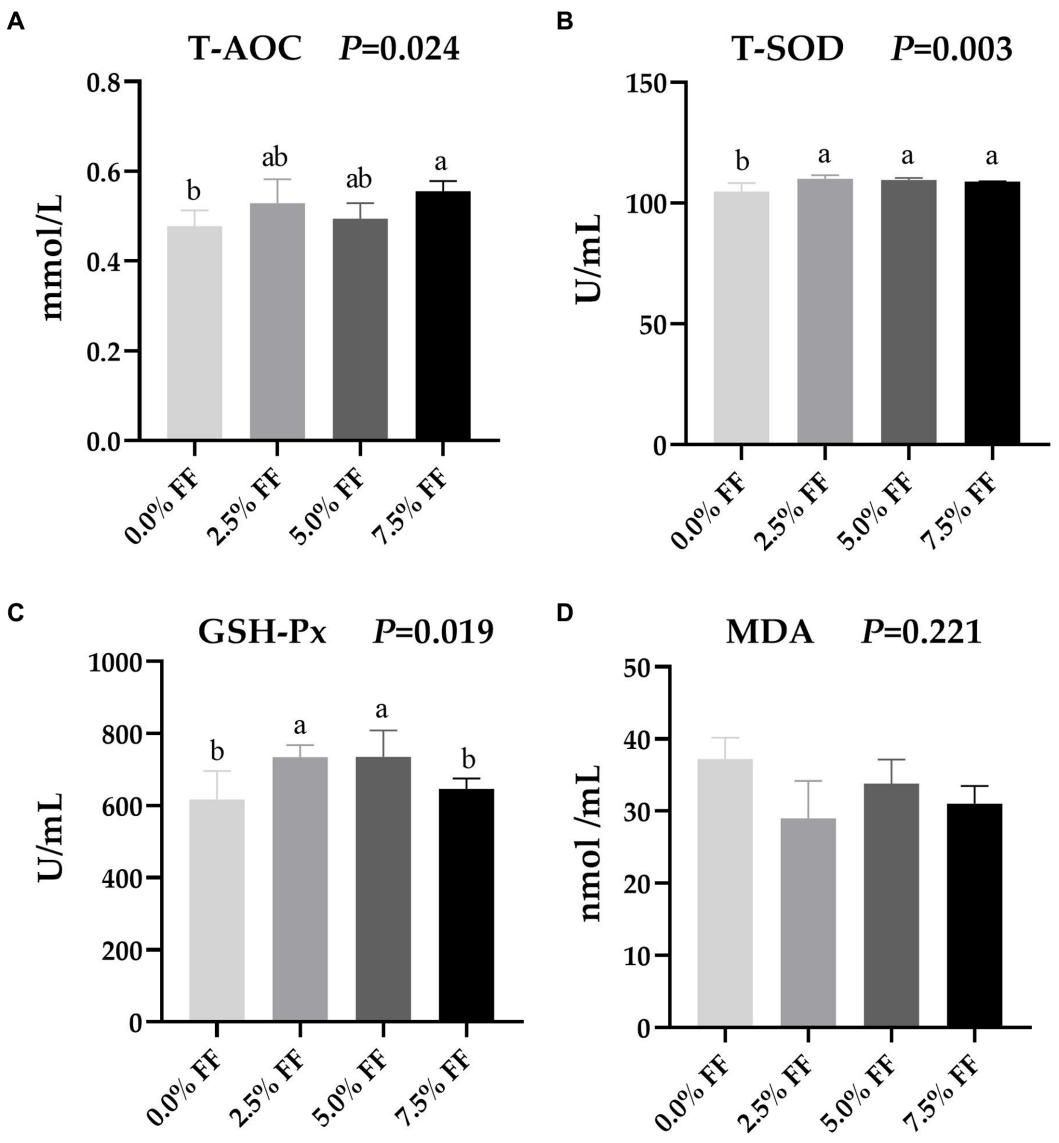
Effects of fermented feed on serum antioxidant function of lion-head goslings. **(A–D)** Refer to activity of serum T-AOC, activity of serum T-SOD, activity of serum GSH-Px and serum level of MDA, respectively. ^a,b,c^ Values with superscripts of different letters in the same row were significantly different (*p* < 0.05), whereas values with the same or no superscripts showed no differences (*p* ≥ 0.05).

According to Figure 1, compared with the control group, serum T-AOC activity were significantly higher in the 7.5% FF group (*p* < 0.05); while the serum T-SOD activity were significantly increased in all FF groups (*p* < 0.05). Moreover, the 2.5 and 5.0% FF groups increased the activity of GSH-Px (*p* < 0.05). However, dietary FF supplementation had no significant on the level of MDA (*p* > 0.05).

### 3.4. Effects of fermented feed on intestine morphology and development of lion-head goslings

**Figure 2:**
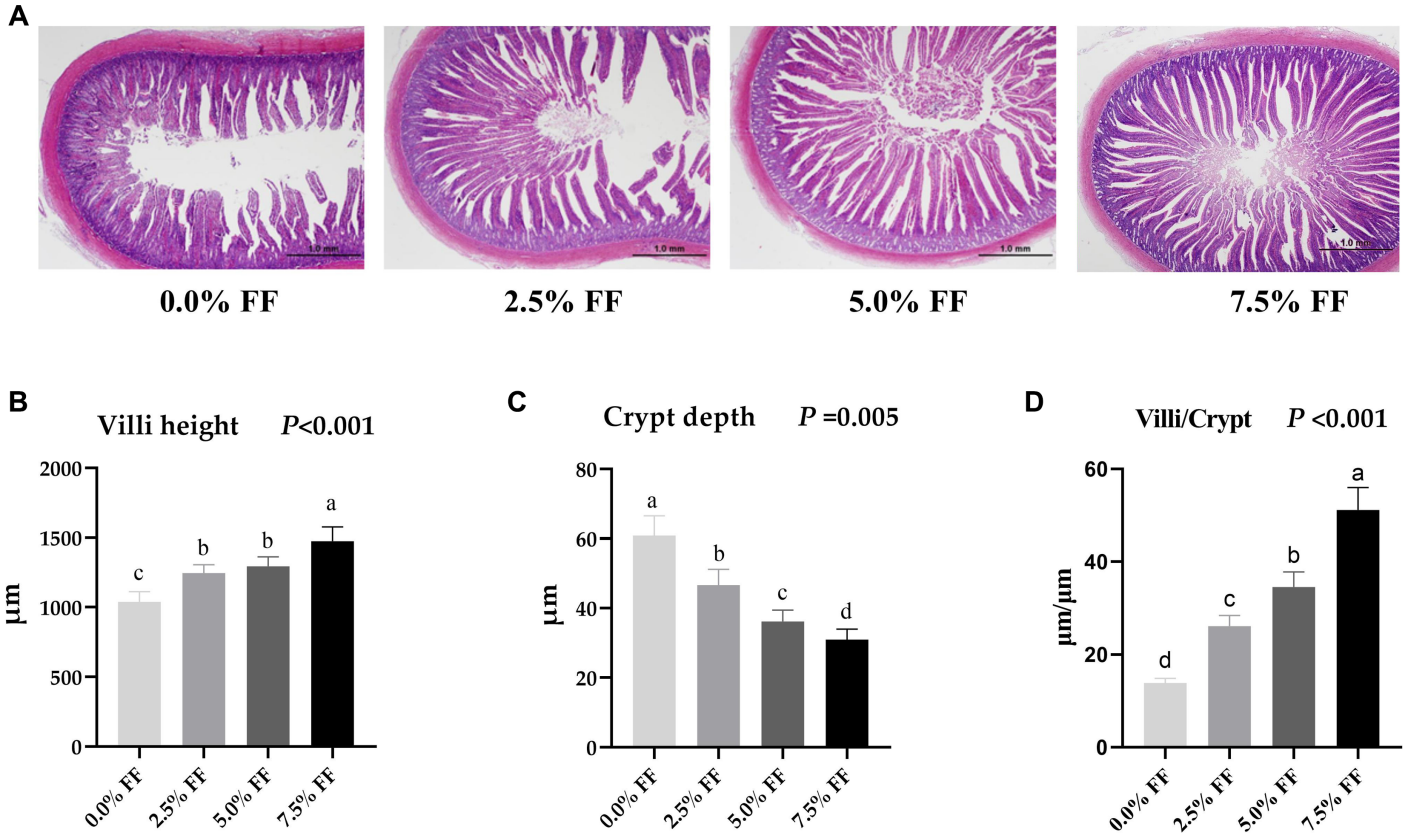
Effects of fermented feed on intestine morphology development of lion-head goslings. **(A)** Measurement of villous height and crypt depth in jejunum; scale length is 1.0 mm. **(B–D)** Refer to villous height, crypt depth and villi/crypt in the jejunum, respectively. ^a,b,c,d^ Row means with different superscripts are significantly different (*p* < 0.05).

As shown in Figure 2, compared with the control group, both the villous height and the villi/crypt ratio in jejunum were increased in all FF groups (*p* < 0.05), where crypt depth decreased significantly with the increase of the FF ratio (*p* < 0.05). Herein, there was no significant difference in jejunum villus height between the 2.5 and 5.0% FF groups (*p* > 0.05). These data reveal that FF has a dose-dependent effect on villi height, crypt depth, and villi/crypt ratio.

### 3.5. Effects of fermented feed on intestinal antioxidant function in lion-head goslings

**Figure 3:**
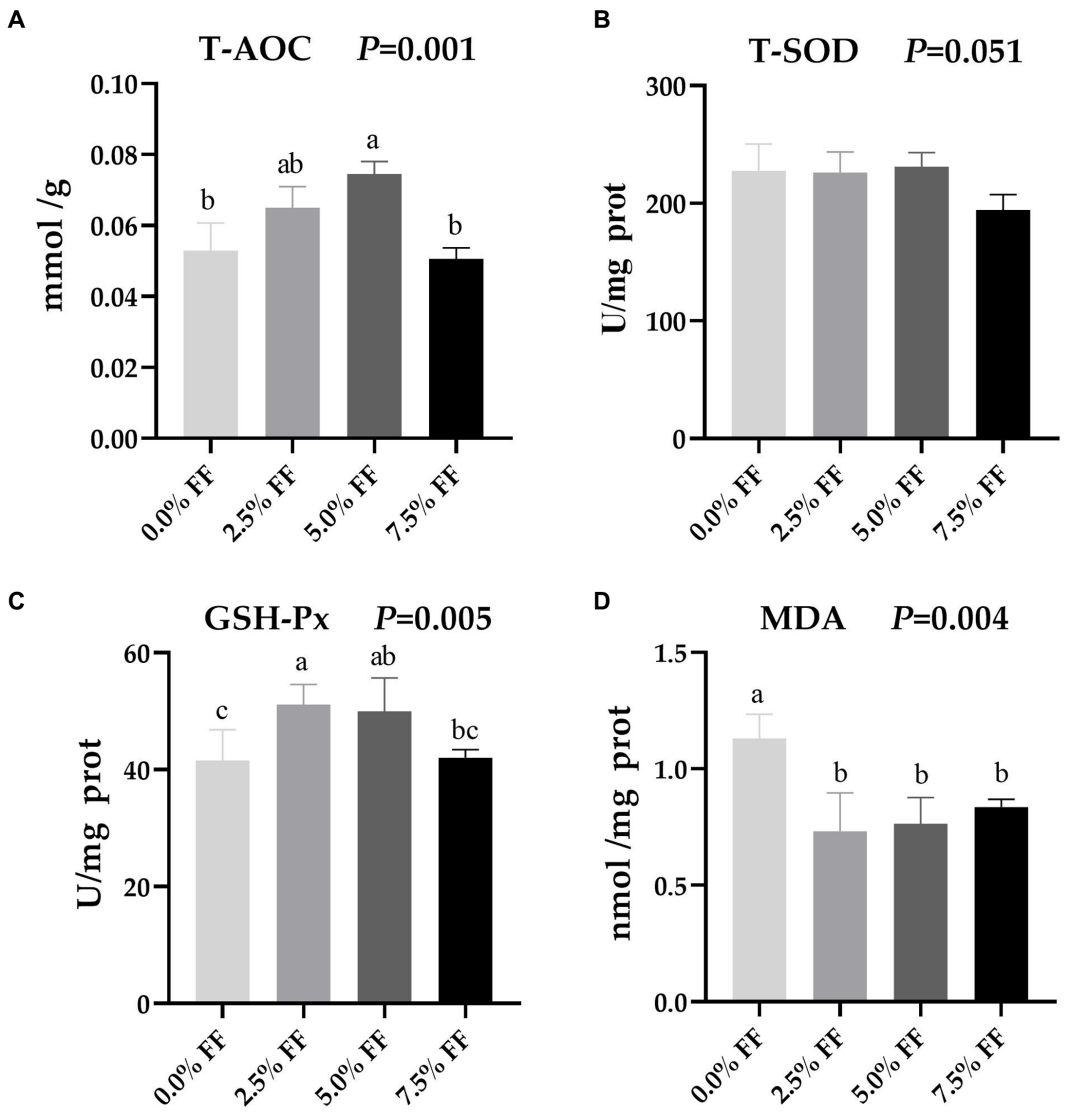
Effects of fermented feed on intestinal antioxidant function of lion-head goslings. **(A–D)** Refer to activity of T-AOC, activity of T-SOD, activity of GSH-Px and level of MDA in jejunum, respectively. ^a,b,c^ Values with superscripts of different letters in the same row were significantly different (*p* < 0.05), whereas values with the same or no superscripts showed no differences (*p* ≥ 0.05).

According to Figure 3, compared with the control group, the 5.0% FF group significantly increased T-AOC activity in the jejunum (*p* < 0.05). Meanwhile, GSH-Px activity in the 2.5 and 5.0% FF groups was significantly increased (*p* < 0.05). However, there was no significant difference between the 5.0 and 7.5% FF groups (*p* > 0.05). Additionally, compared to the control group, MDA levels decreased in all FF groups (*p* < 0.05). There was no significant effect of the treatment groups on T-SOD activity (*p* > 0.05).

### 3.6. Effects of fermented feed on gut microbiota composition and diversity in lion-head goslings

**Figure 4:**
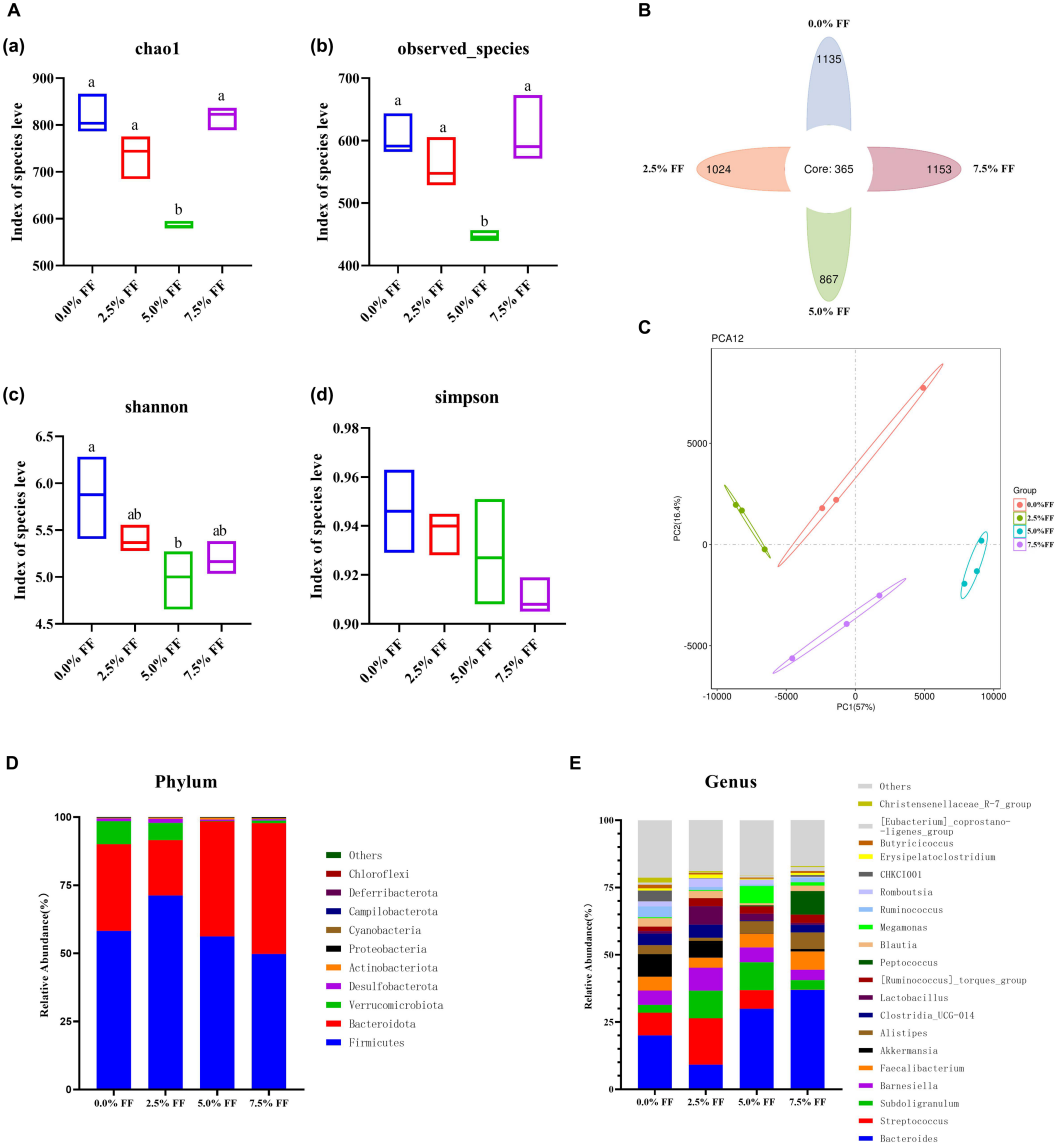
Effects of fermented feed on microbial diversity of lion-head goslings. **(A)** Alpha diversity analysis. **(B)** Venn diagram. **(C)** PCA results. **(D)** Relative abundance of the top 10 phylum-level groups in each test group. **(E)** Relative abundance of the top 20 genus-level groups in each test group.

Chao1, observed species, Shannon index, and Simpson index were used to characterize the alpha diversity of microorganisms in each group of samples. According to Figure 4A, compared with the control group, the 5.0% FF group significantly decreased (*p* < 0.05) chao1, observed species, and Shannon index of cecal microbiota, while the Simpson indexes of all groups were not significantly different (*p* > 0.05). The Venn diagram shows a total of 365 OTUs among the four groups, with 1,135, 1,024, 867 and 1,153 OTUs for each group in order (Figure 4B). The PCA plots showed a significant separation in microbial composition between the treatment groups (Figure 4C).

At the phylum level, the dominant phyla in the cecum contents from the control and FF groups were *Firmicutes*, *Bacteroidota*, and *Verrucomicrobiota*; with *Firmicutes* and *Bacteroidota* accounting for more than 85% of the total population. The relative abundance of cecum contents microbiota increased, whereas the relative abundance of *Verrucomicrobiota* decreased in the 5.0 and 7.5% FF groups compared to the control group (Figure 4D). At the genus level, the dominant genera of cecum contents in the control and all FF groups were *Bacteroides*, *Streptococcus* and *Subdoligranulum*. Compared to the control group, the relative abundance of *Bacteroides* was increased in the 5.0 and 7.5% FF groups, while the relative abundance of *Lactobacillus* was increased in the 2.5 and 5.0% FF groups. Moreover, the relative abundances of *Megamonas* and *Peptococcus* were increased in the 5.0 and 7.5% FF groups, respectively (Figure 4E).

**Figure 5:**
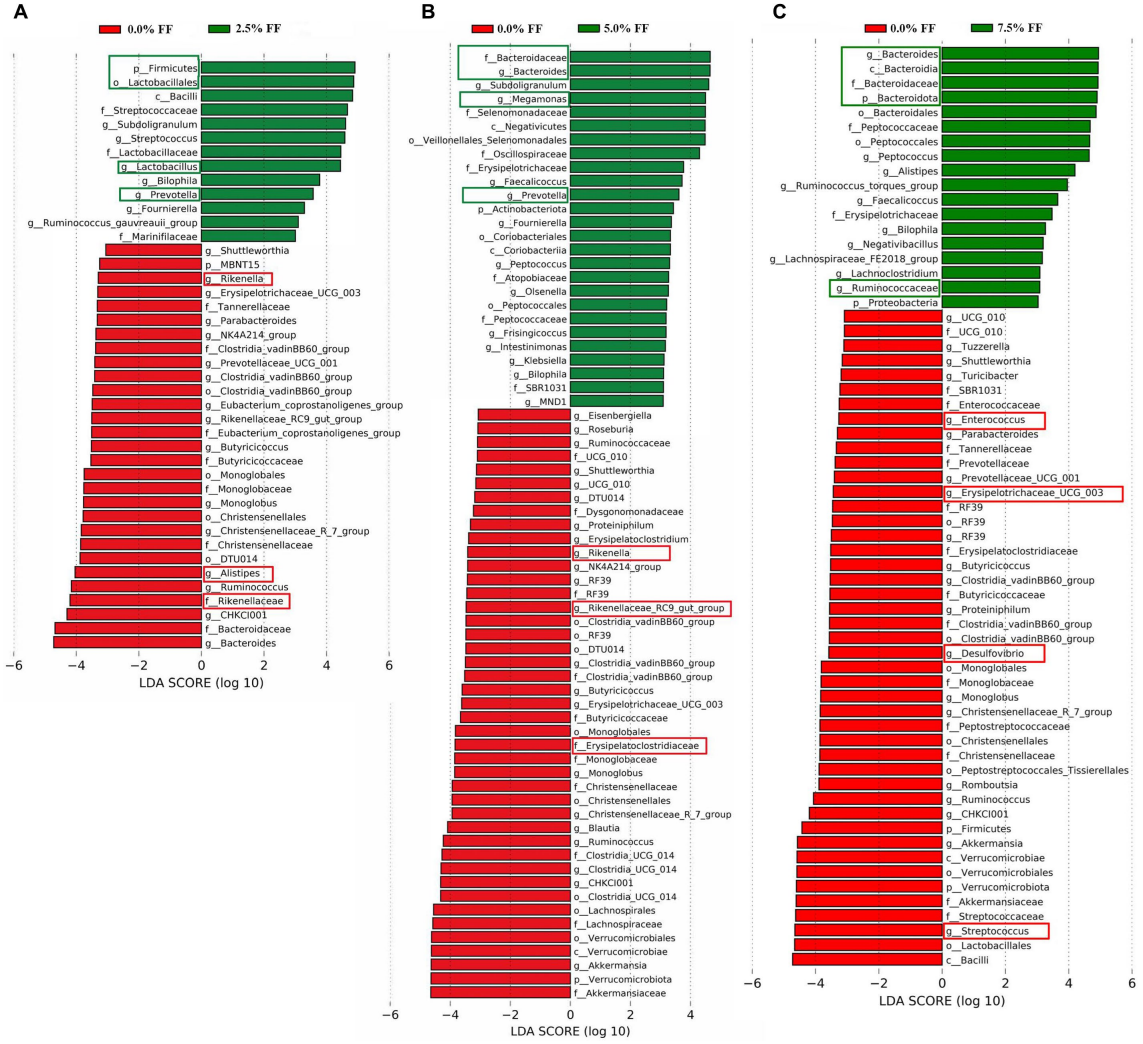
LEfSe histograms, using the default parameter (LDA score > 3.0) identified bacterial taxa with significantly different abundance between all fermented feed groups and the control group **(A–C)** indicating 2.5, 5.0, and 7.5% fermented feed groups versus the control group, respectively.

For the results of LEfSe analysis, when compared to the control group, the significant dominant microflora in the cecum contents of the 2.5% FF group at all levels included *Firmicutes*, *Lactobacillales*, *Lactobacillus*, and *Prevotella*; while the significant dominant microflora in the control group at all levels included *Rikenella*, *Rikenellaceae*, and *Alistipes* (Figure 5A). Compared to the control group, the significantly dominant microflora in cecum contents from 5.0% FF group at all levels included *Bacteroidaceae*, *Bacteroides*, *Megamonas*, and *Prevotella*. The significantly dominant microflora in the control group at all levels included *Rikenella*, *Rikenellaceae*_RC9_gut_group, and *Erysipelatoclostridiaceae* (Figure 5B). The significantly dominant microflora in cecum contents from the 7.5% FF group at all levels included *Bacteroidota*, *Bacteroides*, *Bacteroidaceae*, and *Ruminococcaceae*, while the significantly dominant microflora in the control group at all levels included *Enterococcus*, *Erysipelatoclostridiaceae*, *Desulfovibrio*, and *Streptococcus* (Figure 5C).

## 4. Discussion

FF is gaining increased attention due to their ability to improve the nutrients in feed (probiotics, vitamins, organic acids, amino acids, peptides, enzymes and growth factors, etc.), thus enhancing the growth performance of animals (26, 27). Indeed, Similarly, Zhu et al. (27) reported that the addition of FF in diets led to significantly increased ADG and significantly decreased FCR of 35-day-old laying hens. In this study, the 5.0 and 7.5% FF groups significantly reduced FCR of 28-day-old goslings. In this study, 28-day-old goslings in the 5.0 and 7.5% FF groups displayed significantly decreased FCR. Zhang et al. (28) reported that the addition of yeast to daily feed led to significantly increased ADG and reduced FCR of Sichuan White Geese. It may be due to the fact that fermented feed reduces pH and pathogenic microbial activity in the gastrointestinal tract of the goslings as well as increases the production of short-chain fatty acids (SCFA), which improves intestinal digestibility of the feed, thus improving the growth performance of the goslings (6). However, Wang et al. (29) found that the addition of cottonseed meal to FF in diets had no significant effect on the growth performance of 1- to 21-day-old broilers. Sun et al. (30) reported that the dietary addition of fermented feed with *Bacillus subtilis* as the strain could significantly increase the ADG of Hebei meat geese, and geese had high absorption and utilization of substances such as proteins and crude fibers, whereas lignin, cellulose, hemicellulose and other substances that are not easy to be decomposed into small-molecule carbohydrates were substances that are easy to be absorbed and utilized by the intestinal tract, thus improving the growth performance of meat geese. Moreover, the authors also found a positive correlation between growth performance and nutrient digestibility. Chen et al. (31) reported that the addition of *Bacillus subtilis* and *Saccharomyces cerevisiae* in diets improved weight of broilers, which can be attributed to the fact that *Bacillus subtilis* and *Saccharomyces cerevisiae* FF have increased palatability and result in increased feed intake. Additionally, Niu et al. (32) reported that the addition of fermented *Ginkgo biloba* to the diet significantly increased ADG and ADFI in broilers at 42 days of age. Wizna et al. (33) reported that dietary supplementation with fermented cassava by-products increased the body weight of 28-day-old chicks, which may be attributed to the positive effect of fermented feeds on the growth performance of chicks due to the increase in the levels and digestibility of threonine, lysine, leucine, and methionine in the diet. However, the addition of fermented cassava by-products to the rations had no effect on the growth performance of 28-day-old chicks, probably because of the increase in glucose content due to the cassava by-products (glucose is produced by the *Bacillus amyloliquefaciens* cellulase enzyme in the fermented cassava by-products in the gastrointestinal digestive process) and glucose is counted as metabolizable energy. The results of the above studies are not entirely consistent with our research results, and this may be related to the FF process, bacterial strains, the level of antinutritional factors in the diet, feeding management, and the different growth stages of the experimental animal species, thus leading to differential effects of FF on the growth performance of animals (1).

Serum biochemical indexes can be used to indicate the metabolic functioning in animals (34). Protein anabolism and material transport in the body are related to the activity of serum TP and serum ALB levels (35). In this study, FF had no significant effect on the activity of TP and ALB in serum of goslings, indicating that FF had no effect on protein metabolism and substance transport in goslings. Moreover, a study (36) indicated that serum levels of TG and TC may reflect the lipid metabolism of the body. In this study, serum TC levels were significantly lower in the 7.5% FF group compared to the control group, It may be due to the fact that fermented feeds help to inhibit the activity of 3-hydroxy-3-methylglutacyl coenzyme A reductase, which reduces cholesterol biosynthesis. Moreover, fermented feeds may reduce the level of cholesterol in the blood by the mechanism of increasing the synthesis of bile acids, thus improving the lipid metabolism of the gosling organism (37). ALP is a group of isoenzymes located in the outer layer of the cell membrane, which catalyze the hydrolysis of organophosphates. It is present in different tissues of the body and it is present in decreasing concentrations in the placenta, ileal mucosa, kidney, bone, and liver. In the liver, ALP is cytoplasmic and present in the tubular membranes of stem cells, and more than 80% of serum ALP is released from the liver and bone (38). Serum ALP is a marker of liver damage reflecting diseases of the hepatobiliary system and has been widely used as a predictive tool for cholestatic liver disease (39, 40). LDH is widely distributed in eggs, liver, heart, kidneys and muscles, and is a cytoplasmic oxidoreductase enzyme that indicates damage and oxidative stress in the body. In addition, LDH, which can be used to diagnose certain tumors, is also a test indicator for myocardial infarction and liver lesions where the abnormal increase of serum LDH activity may correlate with cardiac or liver injuries (41). Studies have shown that when a patient has cancer, an increase in the number of cancer cells consumes large amounts of glucose, which is used for energy through glycolysis, increasing LDH activity under anaerobic conditions. In addition, growing cancer cells also destroy other tissues of the body and release the intracellular enzyme LDH into the bloodstream through damaged and dead cells, thus increasing LDH activity in the blood (42). Yeh et al. (43) demonstrated that *B. subtilis* variant N21+, *B. coagulans* variant L12 in FF led to decreased activity of serum LDH in broilers, which could be caused by the decomposition of the fermentation substrate, or by the reduction of antinutrients due to heat treatment during the pelleting process, thus reducing the activity of serum LDH. Additionally, Wang et al. (40) found that the addition of ginkgo FF to the diet results in a significant decrease in serum ALP activity in meat ducks. In this study, the ALP activity of all FF groups decreased significantly, and the 5.0 and 7.5% FF groups significantly decreased serum LDH activity. These findings suggest that FF protected the liver of goslings to some extent.

Poultry have a protective system that controls the production of free radicals and maintains redox balance; when the balance is disturbed, oxidative stress occurs, resulting in serious effects on the health, growth, and development of poultry (44). T-AOC activity reflect the overall antioxidant capacity of an organism, where an increase represents a higher overall antioxidant capacity of the organism. Additionally, GSH-Px and T-SOD are important antioxidant enzymes in the antioxidant system, which protect the organism from damage by eliminating free radicals in the body. On the other hand, MDA, which is the product of lipid and free radical oxidation reactions in the body, can reflect the degree of lipid peroxidation in the animal body and indirectly reflect the degree of cell damage (45, 46). A study by Hu et al. (36) found that the addition of fermented rapeseed meal in the diet led to significantly increased activity of serum T-SOD in 42-day-old broilers. Similarly, Yin and Huang (47) demonstrated that the addition of fermented alfalfa meal (FAM) in the diet led to significantly increased activity of serum GSH-Px and T-SOD in geese, and significantly decreased serum MDA levels. Moreover, it has been shown that antioxidant function is associated with the nuclear factor-erythroid 2-related factor 2 (Nrf2) signalling pathway, which is a major regulator of oxidative stress damage defence mechanisms and plays an important role in cellular defence against a variety of inflammatory and oxidative stress-induced diseases (48, 49). Niu et al. (34) showed that the addition of fermented *Ginkgo biloba* to broiler diets increased the mRNA expression of Nrf2, which ultimately increased GSH-Px activity in serum, and T-AOC and sod activity in liver. In this study, the addition of FF in the diet led to significantly increased T-AOC, T-SOD, and activity of GSH-Px of goslings. This may be due to the production of beneficial metabolites such as vitamins and small peptides after the fermentation process which are then present in the FF. These substances help to balance the production of pro-oxidants and antioxidants in the body, thus improving the serum antioxidant capacity of goslings (50).

The small intestine is the main site of nutrient absorption in the animal body, and its surface folds and villi can greatly increase the area for nutrient absorption. Villus height, crypt depth and the ratio of villus to crypt are important indicators to measure the normal function of intestinal mucosa. Specifically, the higher the villous height, the larger the surface area of the intestine and the stronger the absorption capacity of nutrients; whereas the crypt depth reflects the maturation rate of intestinal epithelial cells and the shallower the crypt depth, the increased number of mature cells; finally the villi/crypt ratio reflects the normal morphology and function of the intestine (26, 36, 51). Ding et al. (52) reported that the addition of solid-state fermented sesame meal in diets led to significantly increased villous height and the villi/crypt ratio in jejunum of 21-day-old meat duck. Similarly, Chiang et al. (26) demonstrated that the addition of solid-state fermented rapeseed meal in diets led to significantly increased villous height and villi/crypt ratio in the jejunum and ileum of 21-day-old broilers. Guo et al. (8) reported that the addition of FF in diets led to significantly increased villous height in the duodenum, jejunum, and ileum of laying hens, which could be attributed to the inhibition of Campylobacter and other pathogens by Lactobacillus, thereby reducing damage to the intestinal mucosal epithelium. The results of the present study were in general agreement with Xie et al. (53), in which the villous height and villi/crypt ratio in jejunum increased significantly in all FF groups, while crypt depth decreased significantly. This suggests that FF has a positive effect on intestinal structure. Furthermore, the improvement of intestinal morphology may be attributed to the increase of gastrointestinal probiotics in the FF. After entering the intestine, probiotics colonize and proliferate, supporting a series of metabolic processes The metabolites produced are subsequently absorbed by mucosal cells and promote the growth of intestinal epithelial cells, thus improving the structure of the empty intestinal mucosa, increasing villous height, and reducing crypt depth (53, 54).

Intestinal oxidative stress has a great impact on the health and production of poultry (32). Reactive oxygen species (ROS) are produced by intestinal epithelial cells through oxygen metabolism or intestinal symbiotic bacteria, which affect intestinal health. Increased ROS will lead to the production of free radicals and antioxidant damage, resulting in intestinal oxidative stress. In addition, intestinal oxidative stress can lead to lipid peroxidation, protein modification, and DNA damage as well as membrane damage, which can hinder efficient digestion and absorption of nutrients. Fortunately, supplementation with exogenous vitamins, antioxidants and plant additives with antioxidant characteristics can scavenge ROS, remove intestinal free radicals, help alleviate intestinal oxidative stress, and maintain the integrity of the intestinal mucosa (55, 56). Czech et al. (57) reported that the addition of fermented rapeseed or/and soymeal in diets led to significantly increased activity of SOD in the jejunum of piglets. Furthermore, Niu et al. (32) found that the addition of fermented ginkgo leaf in diets significantly improved the capacity of T-AOC and activity of GSH-Px in the jejunum of broilers and significantly decreased level of MDA. In this study, the activity of T-AOC in the jejunum from 5.0% FF group increased significantly, and 2.5 and 5.0% FF groups’ activity of GSH-Px increased significantly, and all FF groups showed significantly decreased level of MDA in the jejunum, suggesting that FF can enhance antioxidant capacity, to protect intestinal epithelial cells from oxidative stress. FF may also favor the generation of α-amylase, which is responsible for degradation of the cell wall matrix, thereby releasing polyphenols such as flavonoids, which can regulate the production of ROS and antioxidant in cells, thus improving the antioxidant capacity of goose intestine (58–60).

The gastrointestinal tract of poultry contains a complex and dynamic microbial community, where the balance of gastrointestinal microecology is closely related to the health and growth performance in hosts, and changes in gut microbiota can directly affect the digestion and absorption of nutrients in the animal body (61). The diversity of gut microbiota affects the stability of the microflora and the ability of the host to resist pathogenic bacteria (62). FF help to regulate the composition of the gut microbiota of the animal and maintain a healthy gastrointestinal ecosystem by preventing excessive inflammatory responses to pathogens in the intestine (63).

*Firmicutes* and *Bacteroidota* are the two major flora in the avian cecum, playing an important role in nutrient digestion and absorption and host metabolism (64). *Firmicutes* are involved in the digestion of various types of nutrients, promoting the fermentation of polysaccharides in the intestine and improving the health of the host (62). Li et al. (65) reported the addition of FF in diet and found that cecal microbiota of broilers was dominated by *Firmicutes* and *Bacteroidota*. Yan et al. (14) reported addition of FF in diet of geese and found that cecal microbiota of the 7.5% FF group was dominated by *Firmicutes* and *Bacteroidota*, and the relative abundance of the former is relatively low and the relative abundance of the latter is relatively high, compared with the control group. Our results are in agreement with the previous findings that the high levels of *Bacteroidota* in FF may help to improve the digestibility of nutrients in the intestinal tract of geese (66). *Bacteroidota*, are the second largest gut microbiota phylum in poultry and have propionate and acetate as fermentation end products, which are required by the host and gut microbiota. Furthermore, *Bacteroidota* can degrade macromolecular compounds in the intestinal tract and promote the digestion and absorption of proteins, lipids and polysaccharides in animal body (66, 67). At the genus level, the relative abundance of *Lactobacillus* in both 2.5 and 5.0% of FF groups was increased, with a significant predominance of *Lactobacillus* in the 2.5% FF group. *Lactobacillus* can help prevent the growth and reproduction of pathogenic bacteria in the gastrointestinal tract by lowering the pH of the intestinal environment through the production of lactic acid. Meanwhile, *Lactobacillus* secretes a variety of metabolites such as organic acids, hydrogen peroxide and carbon dioxide, and hydrogen peroxide activates the catalase-thiocyanate system. When lactate peroxidase is combined with hydrogen peroxide and reacts with thiocyanate to produce oxidative intermediates, it can inhibit the growth of pathogenic bacteria (68, 69). LEfSe analysis showed that except for *Bacteroides* and *Bacteroidaceae* in 5.0 and 7.5% FF groups, *Megamonas* and *Prevotella* were the dominant bacteria in the 5.0% fermented diet group, while *Ruminococcaceae* were the dominant bacteria in the 7.5% fermented diet group. In recent studies, *Prevotella* is generally considered a beneficial genus of bacteria, which plays a key role in the carbohydrate metabolism of the body (70). *Ruminococcaceae* is the main family in rumen cellulolytic bacteria, which can degrade macromolecules such as cellulose and hemicellulose into nutrients such as short-chain fatty acids (SCFAs) that can be easily digested and absorbed by animals, thus promoting growth (14, 71, 72). Additionally, *Desulfovibrio* showed a significant advantage in the control group, which included sulfate-metabolizing bacteria, which are known to reduce dietary sulfites as well as sulfated mucopolysaccharides in mucins, leading to the production of the cytotoxic compound hydrogen sulfide, which adversely affects the organism (73). In conclusion, FF can increase the abundance of cecum probiotics, thus regulating the balance of lion-head goslings gut microbiota and improving their overall intestinal health.

## 5. Conclusion

In conclusion, dietary FF supplementation improved growth performance, serum biochemical indexes, antioxidant capacity and intestinal flora structure of lion-head geese. Notably, dietary 7.5% FF supplementation was optimal for the growth and intestinal health of lion-head geese.

## Data availability statement

The datasets generated for this study can be found in the NCBI Repository [accession number: PRJNA1027998].

## Ethics statement

The animal study was approved by the Animal Care Committee of Zhongkai University of Agriculture and Engineering (approval number: 20220321). The study was conducted in accordance with the local legislation and institutional requirements.

## Author contributions

ZF: Data curation, Writing – original draft. NA: Writing – original draft. XL: Investigation, Supervision, Writing – review & editing. JC: Investigation, Supervision, Writing – review & editing. YW: Investigation, Supervision, Writing – review & editing. QW: Conceptualization, Methodology, Writing – review & editing. JF: Project administration, Conceptualization, Methodology, Writing – review & editing. CL: Writing – review & editing. LL: Writing – review & editing.
